# Enhancing the Conservation of Crop Wild Relatives in England

**DOI:** 10.1371/journal.pone.0130804

**Published:** 2015-06-25

**Authors:** Hannah Fielder, Peter Brotherton, Julian Hosking, John J. Hopkins, Brian Ford-Lloyd, Nigel Maxted

**Affiliations:** 1 School of Biosciences, The University of Birmingham, Edgbaston, Birmingham, United Kingdom; 2 Natural England, Unex House, Bourges Boulevard, Peterborough, United Kingdom; 3 Natural England, Riverside Chambers, Castle Street, Taunton, Somerset, United Kingdom; 4 Environment and Sustainability Institute, University of Exeter, Penryn, Cornwall, United Kingdom; National Institute of Plant Genome Research (NIPGR), INDIA

## Abstract

Humans require resilient, rapidly renewable and sustainable supplies of food and many other plant-derived supplies. However, the combined effects of climate change and population growth compromise the provision of these supplies particularly in respect to global food security. Crop wild relatives (CWR) contain higher genetic diversity than crops and harbour traits that can improve crop resilience and yield through plant breeding. However, in common with most countries, CWR are poorly conserved in England. There is currently no provision for long-term CWR conservation *in situ*, and comprehensive *ex situ* collection and storage of CWR is also lacking. However, there is a commitment to achieve their conservation in England’s Biodiversity Strategy and the UK has international commitments to do so as part of the Global Plant Conservation Strategy. Here, we identify a series of measures that could enhance the conservation of English CWR, thereby supporting the achievement of these national and international objectives. We provide an inventory of 148 priority English CWR, highlight hotspots of CWR diversity in sites including The Lizard Peninsula, the Dorset coast and Cambridgeshire and suggest appropriate sites for the establishment of a complementary network of genetic reserves. We also identify individual *in situ* and *ex situ* priorities for each English CWR. Based on these analyses, we make recommendations whose implementation could provide effective, long-term conservation of English CWR whilst facilitating their use in crop improvement.

## Introduction

The most recent report by the Intergovernmental Panel on Climate Change (IPCC) suggests that crop yields will decrease by an average of 2% per decade due to the negative impacts of climate change; with more severe forecasts expected beyond 2050 [1]. This worrying statistic is compounded by another equally concerning prediction that the rise in the human population over the next 90 years [2] will require global food production to increase by up to 70% [3,4]. In order to ensure future food security, not only will crop yields need to increase significantly but the crops themselves will need to become progressively more resilient to changing conditions. As a result, plant breeders are now looking to crop wild relatives (CWR) and, more specifically, the largely untapped gene pool of genetic diversity within them as the key to tackling these issues through conventional plant breeding [5].

CWR are the wild plants related to crops of socio-economic value, such as human food and animal forage and fodder crops as well as those used for medicinal, forestry, industrial and ornamental purposes etc. Though the conservation of CWR related to all plant-derived supplies is strongly encouraged, the focus of this paper is in the context of CWR related to human food and animal forage and fodder crops in order to address the pressing issue of food security. In contrast to their cultivated relatives, CWR have not passed through the genetic bottleneck of domestication [6]. As such, CWR harbour higher levels of genetic diversity and potentially contain a range of traits that could be used for crop improvement to increase the resilience and yield of modern crop varieties. The closeness of the relationship between a crop and its CWR can be defined in terms of the Gene Pool (GP) concept [7], where genes in CWR belonging to the primary Gene Pool (GP1b) of a crop can easily be transferred to the crop (belonging to GP1a). CWR in the secondary gene pool (GP2) can be crossed with the crop with some success but CWR in the tertiary gene pool (GP3) require biotechnological approaches to facilitate gene transfer [7]. However, gene pool studies are often lacking, particularly for less studied crops, and so the Taxon Group (TG) concept relying upon traditional taxonomic analyses of relatedness can be employed to define this relationship [5]. In this case, TG1a corresponds to the crop/GP1a, TG1b denotes CWR belonging to the same species as the crop, TG2 denotes CWR belonging to the same section as the crop, TG3 being those belonging to the same subgenus and finally TG4 being those belonging to the same genus as the crop. Those CWR where gene transfer to a related crop is possible can contribute significantly to improving crop varieties, and their use in this way will become increasingly important [8,9]. An extensive literature exists detailing examples of the use of CWR in crop improvement [10]. The introduction of *Cercospora* leaf spot and Rhizomania resistances from wild sea beet (*Beta vulgaris* L. subsp. *maritima* (L.) Arcang.) into cultivated sugar beet [11,12], the transfer of corn leaf blight resistance from wild *Tripsacum dactyloides* (L.) L. into maize [13] and more recently, the identification of the potential for wild barley (*Hordeum vulgare* L. subsp. *spontaneum* (K. Koch) Thell.) to improve the drought tolerance of cultivated barley [14] are just a handful of examples of the use of CWR in plant breeding programmes for crop improvement. Recent estimates suggest that the potential for contribution of beneficial traits from CWR for 29 priority crops identified by the Millennium Seed Bank, Kew (including wheat, rice and potato) could alone amount to approximately $120 billion [15].

Despite the recognition of their potential value in crop development, CWR are also highly threatened by factors which are impacting all wild plant species, such as the effects of habitat destruction, nutrient enrichment and climate change [16]. In Britain it has been estimated that an average of one wild vascular plant was lost per county every two years throughout the twentieth century [17]. This decline can be taken as a proxy for CWR decline, but worryingly CWR remain poorly conserved both *in situ* and *ex situ* globally and in the UK [9,18,19,20]. This has led to a call for improved conservation of genetic resources such as CWR [9,21], working towards safeguarding their populations and the range of genetic diversity contained within.

The initial step required to improve CWR conservation is to identify which CWR taxa require improved protection. This can be achieved through the creation of a CWR checklist and inventory. A checklist simply consists of names of taxa and their authorities within the geographical region of study [22]. It is likely to be unrealistic to conserve all CWR taxa in a checklist due to limitations of resources, time and money. Hence a prioritisation process is required, selecting appropriate criteria in order to create an inventory of CWR taxa. A CWR inventory consists of a prioritised list of CWR with ancillary information for each taxon [22]. Production of CWR inventories is essential in development of conservation strategies, allowing a more focussed approach that targets taxa of highest priority in a given region of study [23,24].

Early European CWR checklists (e.g. by Zeven and Zhukovsky [25], containing 430 plant genetic resource (PGR) species; Heywood and Zohary [26], containing 206 CWR species and subspecies) tended to focus solely on the primary gene pools of the globally most important cultivated crops [27]. More recently a Crop Wild Relative Catalogue for Europe and the Mediterranean was created, an inclusive checklist of 25,687 crop and CWR taxa occurring within Europe and the Mediterranean [28,29]. As noted by Maxted *et al*. [27], this checklist takes a more comprehensive view of CWR as it includes all European CWR taxa related to any socio-economically important crops, not just those used in food production. It also enables the extraction of CWR checklists for each European country via the Crop Wild Relative Information System, CWRIS [28].

Increasing numbers of national inventories of priority CWR are now being developed to encourage CWR conservation worldwide. Recently, prioritised CWR inventories for Venezuela, Benin and the USA have been developed [30–32]. In Europe, CWRIS has been used as a starting point for the creation of a national inventory of CWR for Portugal [33] as well as inventories for Finland, Spain, Italy and Cyprus [34–37]. Although all of these inventories have been developed in a similar manner there are key differences in the approaches taken, particularly in terms of the criteria used in prioritising CWR. The selection of appropriate criteria is largely dependent on the specific requirements of the geographical region of study, those undertaking the inventory and the available information on which to base the prioritisation.

Once priority CWR have been identified for any geographical area of study it is then necessary to carry out a ‘gap analysis’. This is a process whereby the extent of current conservation efforts for priority taxa are examined and decisions made as to where further conservation efforts are necessary to ensure the long-term persistence of populations and the genetic diversity within them, using both *in situ* and *ex situ* approaches [38]. A number of gap analyses have now been successfully carried out for a range of CWR taxa in diverse locations. This has led to the identification of conservation priorities for wild soybean (*Glycine* Willd.) relatives in Australia [39], beans (*Vigna* Savi) in Africa [40], beans (*Phaseolus* L.) throughout North, Central and South America [41], and national conservation priorities for Finland, Spain, Italy and Cyprus [34–37].

Despite significant progress being made across Europe, no conservation priorities for CWR in the UK have yet been identified. Approximately eight percent of European CWR occur within the UK [29]. These include wild relatives of economically important crops such as sugar beet and *Brassica* crops (e.g. cabbage, broccoli and Brussels sprouts) which are of particular commercial interest and which highlight the international role that the UK can play in the conservation of CWR [9].

Previous CWR inventories for the UK have predominately consisted of short lists of minor crops with wild UK populations rather than the wild relatives themselves [27,42,43]. Since then Defra [44] have committed to conserving “agricultural genetic diversity in cultivated plants, farmed animals and wild relatives” as part of their Biodiversity 2020 strategy for England. This follows on from commitments to the Convention on Biological Diversity’s Global Strategy for Plant Conservation as well as target 13 of the Strategic Plan for Biodiversity which states that ‘by 2020, the genetic diversity of cultivated plants and farmed and domesticated animals and of wild relatives, including other socio-economically as well as culturally valuable species, [will be] maintained, and strategies [will] have been developed and implemented for minimizing genetic erosion and safeguarding their genetic diversity’ [45–47]. This is reiterated in the European Strategy for Plant Conservation [48] and Europe’s own Biodiversity 2020 strategy [49]. Such policy documents are now providing the impetus for national conservation organisations within the UK to move genetic conservation of CWR higher up their agenda.

The objective of the current study was to support the achievement of these national and global commitments to conserve CWR in England. Conservation within the UK is managed separately within each of the devolved administrations of the UK, with some aspects coordinated by the Joint Nature Conservation Committee (JNCC). As such it was deemed appropriate to devise conservation priorities for England, Wales and Scotland separately with the involvement of the relevant statutory organisations in each country (Fielder *et al*., in prep a,b). In this way it could be ensured that the priorities identified were supported in each respective country. The University of Birmingham and Natural England (NE) developed the conservation priorities identified in the current study for English CWR jointly. Targets for CWR conservation are here identified and recommendations made as to how systematic, active and long-term conservation can be achieved in England to secure these valuable resources.

## Materials and Methods

### CWR checklist and inventory

A checklist of English CWR was developed by matching a UK checklist of CWR (derived from CWRIS [27,28] with a checklist of the English flora, extracted from the Vice County Census Catalogue (VCCC) [50]. The taxonomy in the English CWR checklist was then standardised against the British flora [51]. The final checklist contains 1471 CWR taxa including those related to all socio-economic crops (e.g. human food, animal forage and fodder, medicinal, forestry, industrial, ornamental). The CWR checklist includes native, archaeophyte, neophyte and casual taxa and represents 35% of the total English flora (again, including all native and introduced taxa). The checklist is available at the Plant Genetic Resources Diversity Gateway (http://pgrdiversity.bioversityinternational.org).

The usual approach to identifying conservation priorities for vascular plants (e.g. considering rarity, threat and rate of decline) is not appropriate in the case of CWR. For CWR the focus is shifted towards those taxa that are the most useful or valuable in terms of their potential contribution to developing improved crop varieties, and in this particular case, food security. As a result, it is criteria such as the economic value of the crop with which they are related and the closeness of the genetic or taxonomic relationship between CWR and crop that infer which CWR should be considered a priority for conservation. Prioritisation of the CWR checklist was based on five criteria listed below, the selection of which aimed at the identification and conservation of genetic resources most likely to support resilience in global food production. Criteria one and two were first applied to the CWR checklist to identify a pool of native and archaeophyte CWR that were most relevant to food security considerations. The remaining three criteria (criteria 3 to 5) were applied in turn to this CWR pool. A CWR was required to meet the conditions of just one of criteria 3 to 5 to be listed in the final inventory of priority CWR for England.

1. Use of the related crop—CWR related to crops used as a human food source or for animal feed (forage/fodder) were prioritised due to their being the CWR most relevant to ensuring future food security. ‘Use’ data were extracted from GRIN Taxonomy for Plants [52].
2. Native status—Priority was assigned to taxa classified as native or archaeophyte in England. Data regarding native status was extracted from the VCCC [50].
3. Economic value of the related crop—A list of economically important human food crops was generated by extracting crop production quantity data (million tonnes) at the global, European and UK levels between 2007 and 2011 from FAOSTAT [53], crop production value at producer price data between 2007 and 2011 (millions of euro) from Eurostat [54] and crop production at market prices data (£ million) at the UK level between 2006 and 2010 from Defra [55]. Data representing a period of five years were extracted to reflect recent value and current trends in agriculture. However, when long-term trends were considered (period of 20 years), identical crop lists were obtained. Wild relatives of all crops with data relating to any of these statistics were prioritised. Equivalent economic values for forage and fodder crops were unavailable; a similar problem was experienced by Kell *et al*. [16].
4. Degree of relatedness to the crop—Using the Gene Pool and Taxon Group concepts described above, priority was assigned to taxa in GP1b and GP2 and TG1b, TG2 and TG3 of their related crop. Where sub-generic taxonomic classifications were not available, CWR were assigned to TG4. Taxa only occurring in GP1a or TG1a were not prioritised as they are the cultivated forms of the taxa for which wild populations were not recorded as present in England. Data were extracted from the Harlan and de Wet inventory of globally important CWR taxa [56]. It was commonly observed that a single CWR would be related to more than one crop. In these cases, the highest GP/TG that it belonged to was selected, and prioritisation based on this number.
5. Recent change in population range—Priority was assigned to CWR whose population ranges have declined between two recent survey years (1987 and 2004) according to Change Factor (CF) data. CF data take into account the differing range sizes of each species and corrects the data to enable interspecific comparisons to be made [57]. Any CWR with a negative CF value (i.e. below the threshold of zero) indicated population decline and was prioritised.

### *In situ* gap analysis

Occurrence data records for taxa in the final English inventory of priority CWR were extracted from the BSBI distribution database [58]. These raw data were filtered to produce a ‘clean’ dataset of records. Records listed as ‘doubtful’ or ‘unconfirmed’ were excluded, as were any records dated older than 1970. Records lacking both coordinates and location descriptions were removed from the dataset and all records that had a precision lower than tetrad level (2km^2^) were also excluded. The reliability of the final gap analysis results is directly related to the accuracy and quality of data input into the analysis [38,41]. The coordinates for all occurrences in the final dataset were recorded in decimal degrees for compatibility with mapping software.

*In situ* gap analysis was undertaken using the mapping softwares ArcMap 10.0 and DIVA-GIS 7.5.0 [59,60]. Country boundary files were obtained from DIVA-GIS (www.diva-gis.org). Using methods described by Hijmans *et al*. [60] and Scheldeman and van Zonneveld [61] the following GIS functions were carried out:

- Taxon richness and observation richness—to determine hotspots of taxon diversity and to identify any sources of data bias, using the ‘point to grid’ function in DIVA-GIS with a grid cell size of 0.1 degrees.
- Complementarity analysis—using the ‘reserve selection’ function in DIVA-GIS to select potential sites for CWR genetic reserves. An iterative method was used where the first selected site contains the highest number of taxa, the second site was selected on the basis that it contained the next highest number of taxa excluding those contained in the first site etc. [62]. A grid cell size of 0.1 degrees was used. Complementarity results were then further explored to determine which taxa are represented in five or more complementary grid squares. Conserving at least five geographically distinct populations of a taxon decreases the likelihood that it will be lost in the face of stochastic change or through human influence [63,64].
- Identification of *in situ* conservation actions required for each priority CWR—to identify the extent to which CWR taxa are passively conserved *in situ* using spatial join tools in ArcMap 10.0. Boundary shape files for protected areas were obtained from NE [65]. These included: Sites of Special Scientific Interest (SSSI), Special Areas of Conservation (SAC), Areas of Outstanding Natural Beauty (AONB), National Nature Reserves (NNR), Local Nature Reserves (LNR), National Parks, Country Parks, Ramsar sites, Special Protection Areas (SPA) and Biosphere Reserves. Each CWR was then categorised into priority levels according to how well represented they are in protected areas.
  - Priority 1—Poorly represented in protected areas (Less than five protected areas contain five or more occurrence records of the CWR)
  - Priority 2—Poorly represented in SSSIs but well represented in other protected areas i.e. SACs, AONBs, NNRs, LNRs, National Parks, Country Parks, Ramsar sites, SPAs and Biosphere Reserves (Less than five SSSIs contain five or more occurrence records of the CWR but five or more other protected areas contain at least five occurrence records)
  - Priority 3—Well represented in SSSIs (Five or more SSSIs contain at least five occurrence records of the CWR)

For each priority level, recommendations were made for improved *in situ* conservation of the CWR (S1 Table). CWR listed under Section 41 (S41) of the 2006 Natural Environment and Rural Communities (NERC) Act of rare and threatened species were also identified [66].

### *Ex situ* gap analysis

Accession data for priority English CWR were obtained from the UK National Plant Inventory [67] and the Millennium Seed Bank, Kew. Data from these two sources were combined and any accessions lacking data for latitude and longitude fields were georeferenced where possible by comparing their written location description with both the UK Grid Reference Finder (http://www.gridreferencefinder.com) and the Gazetteer of British Place Names (http://www.gazetteer.org.uk).

In order to carry out an *ex situ* gap analysis, the following steps were carried out:

- CWR were listed according to the number of *ex situ* accessions stored in gene banks. A minimum threshold was set at five stored accessions [63,64], above which CWR are considered sufficiently represented in *ex situ* collections but below which further collection is required. A minimum of five accessions was deemed more practical and achievable in the field than other more ambitious thresholds e.g. Brown and Marshall’s recommendation to collect accessions from a minimum of 50 populations [68].
- For all taxa with accession data, the geographic coverage of the accessions and occurrences were compared for each taxon. Using the ‘circular area statistic’, a geographical representativeness score (GRS) was calculated for each taxon (GRS is the proportion of occurrence data covered by accession data for each taxon, expressed as a percentage) [41]. The lower the GRS value, the higher the taxon in terms of its level of priority for collection. A GRS of 30% or less is generally viewed as a threshold, below which further *ex situ* collection of the taxon is advisable [37,41].
- Based on these two *ex situ* results, CWR were categorised into *ex situ* priority levels, 1–6, according to the number of accessions stored *ex situ* per taxon and the GRS value. The highest priority being assigned to CWR for which there are currently no accessions. Conservation actions were recommended according to each priority category (S1 Table).
  - Priority 1—No accessions
  - Priority 2—Has accessions but none are georeferenced/location data restricted
  - Priority 3—Fewer than five accessions and GRS lower than 30%
  - Priority 4—Fewer than five accessions but GRS greater than 30%
  - Priority 5—Greater than or equal to five accessions but GRS lower than 30%
  - Priority 6—Greater than or equal to five accessions and GRS greater than 30%

## Results

### CWR inventory

The English national inventory of priority CWR contains 148 taxa (126 species and 22 subspecies), representing 10% of the taxa listed in the English CWR checklist. A summary of the inventory is displayed in Table 1 (and see full inventory at the Plant Genetic Resources Diversity Gateway, http://pgrdiversity.bioversityinternational.org). Of the 148 priority CWR, 76% are related to food crops whilst the remaining 24% are related only to forage or fodder crops. The English inventory contains 13 plant families, with Poaceae, Brassicaceae and Fabaceae containing the most genera (16, 7 and 7 respectively). The three genera with the highest taxon richness are *Trifolium* L. (clovers, 18 taxa), *Vicia* L. (vetches, 12 taxa) and *Chenopodium* L. (goosefoots, 11 taxa).

**Table 1:** Summary of inventory containing 148 priority CWR in England.

Family	Genera	Species	Infra-specific taxa	Native status
Apiaceae	3	2	4	N
Asteraceae	2	4		A & N
Brassicaceae	7	9	4	A & N
Chenopodiaceae	3	13	1	A & N
Corylaceae	1	1		N
Ericaceae	1	5		N
Fabaceae	7	38	5	A & N
Geraniaceae	1	1		N
Grossulariaceae	1	3		N
Liliaceae	2	9		A & N
Linaceae	1	2	1	N
Poaceae	16	28	4	A & N
Rosaceae	6	11	3	A & N
**Totals**	**51**	**126**	**22**	

(A = Archaeophyte, N = Native).

Through the application of criteria for prioritisation of CWR, 34 food crops with native or archaeophyte CWR within England were of economic value based on agricultural statistics [53,54,55], including sugar beet, barley, onions, apples and various brassica crops. Crops of economic value according to production at market prices data from Defra [55] are illustrated in S1 Fig. All of the native and archaeophyte CWR in England associated with these 34 economically valuable crops were listed in the English inventory of priority CWR (77 CWR in total). In terms of the genetic relationships between priority CWR and their associated crops, little over a quarter of taxa (26%) had available Gene Pool classifications. Within this, 16.89% were classified as GP1b and just 5.41% and 4.05% in GP2 and GP3 respectively. The remaining taxa (74%) were classified using the Taxon Group concept.

Almost half (43%) of English priority CWR had a negative CF showing that their populations have declined between surveys carried out between 1987 and 2004. The most extreme changes are in white clover (*Trifolium repens* L., -84), annual meadow-grass (*Poa annua* L., -68) and wood vetch (*Vicia sylvatica* L., -52). In contrast only 28% were shown to be increasing and a further 28% had no available data. According to the newly published Vascular Plant Red List for England [69], 14% of priority CWR taxa in England are threatened. Both upright goosefoot (*Chenopodium urbicum* L.) and alpine cat’s-tail (*Phleum alpinum* L.) are listed as being Critically Endangered with a further five taxa listed as Endangered and 14 listed as Vulnerable (Table 2).

**Table 2:** Threatened taxa listed in the English CWR inventory.

Taxon	Red List Status	Criterion
*Chenopodium urbicum* L.	CR	A2c AOO trend
*Phleum alpinum* L.	CR	D
*Chenopodium vulvaria* L.	EN	A2c AOO trend
*Lactuca saligna* L.	EN	B1ac(iv) + B2ac(iv)
*Chenopodium murale* L.	EN	A2c AOO trend
*Pyrus cordata* Desv.	EN	D
*Trifolium bocconei* Savi	EN	A2ac AOO trend; D
*Asparagus prostratus* Dumort.	VU	D1
*Apium inundatum* (L.) Rchb.f.	VU	A2c AOO and EOO trend
*Cichorium intybus* L.	VU	A2c AOO trend
*Trifolium fragiferum* L.	VU	A2c AOO trend
*Trifolium ochroleucon* Huds.	VU	A2c AOO trend
*Vicia lutea* L.	VU	A2c AOO trend
*Vicia orobus* DC.	VU	D1
*Allium sphaerocephalon* L.	VU	D1; D2
*Chenopodium bonus-henricus* L.	VU	A2c AOO trend
*Chenopodium glaucum* L.	VU	A2c AOO trend
*Hordeum marinum* Huds.	VU	A2c AOO trend
*Medicago minima* (L.) Bartal.	VU	A2c AOO trend
*Trifolium strictum* L.	VU	D2
*Vicia parviflora* Cav.	VU	A2c AOO trend

VU = Vulnerable; EN = Endangered; CR = Critically Endangered; A2c = reduction in population size based on trend in Area of Occupancy (AOO) or Extent of Occurrence (EOO); B1ac(iv) = EOO less than 5000km^2^ and highly fragmented or in no more than 5 locations and extreme fluctuations in number of locations; B2ac(iv) = AOO less than 500km^2^ and highly fragmented or in no more than 5 locations and extreme fluctuations in number of mature individuals; D = restricted population size (less than 50 mature individuals if CR and less than 250 mature individuals if EN); D1 = Very restricted population of less than 1000 mature individuals; D2 = Very restricted population based on Area of Occurrence or number of locations). Data Source: [69].

### *In situ* gap analysis

*Pastinaca sativa* L. subsp. *sylvestris* (Mill.) Rouy & E. G. Camus was the only priority CWR in England with no occurrence records of sufficient quality, despite being listed as native in the British flora [70]. It is likely that records for this taxon have, until now, been treated as wild parsnip (*Pastinaca sativa* L.) for which there are many more occurrence records (3763). Occurrences recorded only to species level may belong to other subspecies and varieties. For this reason, the inclusion of all *P*. *sativa* records could have introduced inaccuracies to the dataset. This highlights the importance of up-to-date and specific recording to the sub-specific level. In total, 679,521 occurrence data points relating to 147 taxa were included in the *in situ* gap analysis. Small cranberry (*Vaccinium microcarpum* (Turcz. ex Rupr.)) and eastern parsnip (*Pastinaca sativa* L. subsp. *urens* (Req. ex Godr.) Čelak) were found to have the fewest occurrence records with just one each, (again, the latter is likely to have been recorded as *P*. *sativa*), whereas cock’s foot (*Dactylis glomerata* L.) was found to have the highest number of occurrence records (28,793).

**Fig 1:**
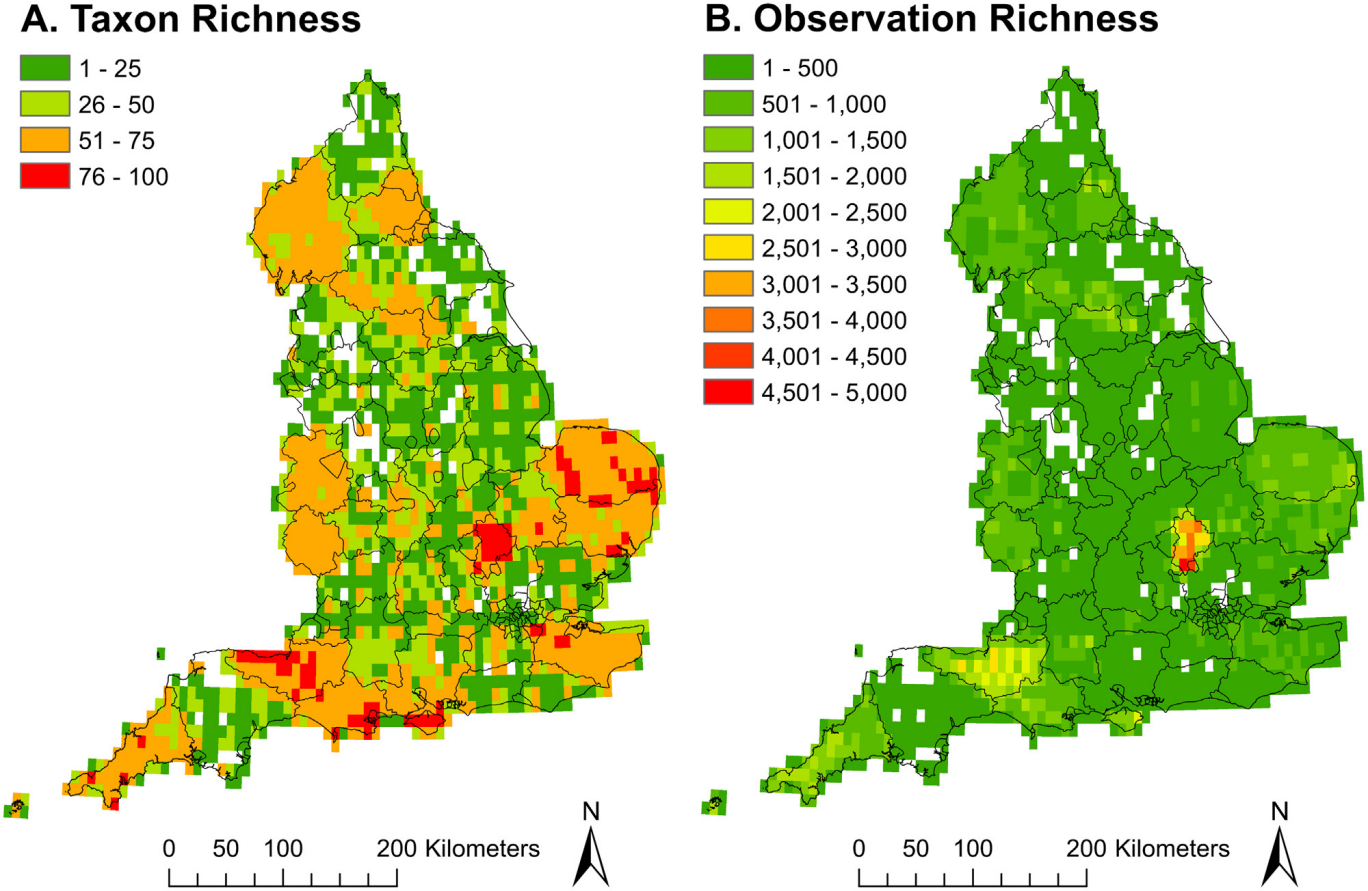
Richness analysis. (a) Taxon richness. (b) Observation richness. Both (a) and (b) include all 147 taxa with occurrence data points in the English national CWR inventory and a grid square size of 0.1 degrees.

Taxon richness analysis of the 147 CWR taxa revealed a number of CWR hotspots throughout England, particularly focussed in the south and east of the country (Fig 1a). These include sites in Cornwall, the Dorset coast, Somerset, Norfolk and Bedfordshire. However, some recording bias is apparent in Bedfordshire (Fig 1b). A new flora was published for this county in 2011 [71], which could account for the recording bias detected in this area.

**Fig 2:**
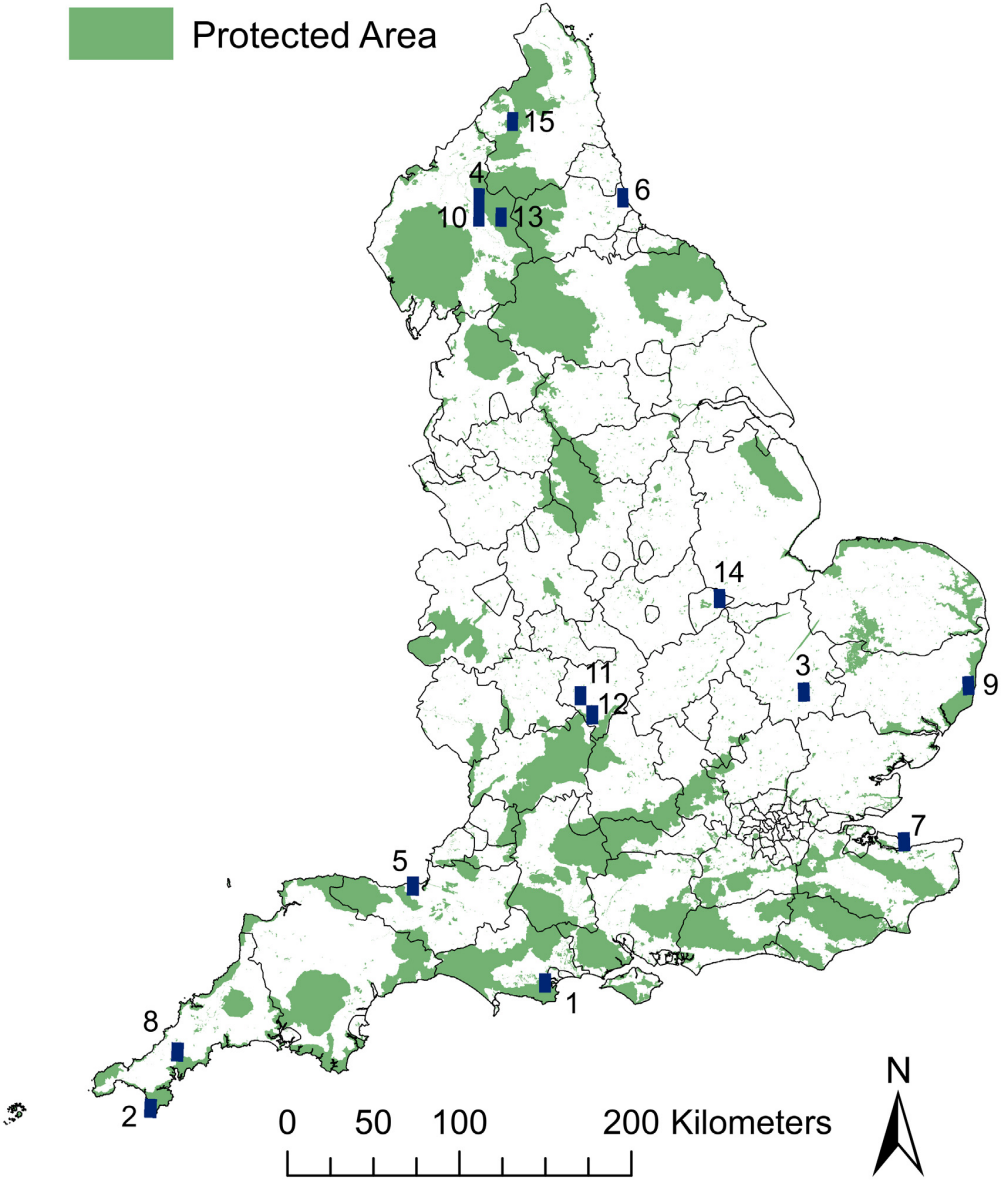
Complementarity analysis. The locations of all 15 priority grid squares/candidate sites recommended for CWR genetic reserves.

The complementarity analysis shows that a total of 15 grid squares (each measuring 0.1 degrees square or approximately 11km^2^) are sufficient to contain at least one occurrence of all priority CWR taxa included in the analysis (Fig 2). The highest priority grid square, containing the highest number of CWR (94), is located in Purbeck on the south coast of Dorset. The second and third priority grid squares are located on The Lizard Peninsula in Cornwall and in the south of Cambridgeshire respectively. The Lizard grid square contains 75 CWR, 14 of which do not occur in the highest priority grid square. The grid square in Cambridgeshire contains a total of 80 CWR, 10 of which do not occur in either of the preceding grid squares (Dorset and The Lizard). Together, the top three priority grid squares cover over 80% of all English priority CWR. The percentage of additional CWR contributed by each grid square is illustrated in S2 Fig. Other priority grid squares were located across the full range of the country, from Cornwall through the midlands to Cumbria and Northumberland. All 15 grid squares overlap with a range of protected areas including SSSIs, AONBs and NNRs. However, these designations do not necessarily provide any protection or active conservation for CWR. In addition, it was found that 53% of priority CWR were recorded in at least five of the 15 complementarity grid squares.

*In situ* conservation actions required for each priority CWR were identified by assigning a priority level to each CWR. Results show that 34 CWR (23%) are poorly represented in protected areas in England (Priority 1) highlighting the need for enhanced conservation for these taxa. An additional 32 CWR (22%) are poorly represented in SSSIs but are well represented in other protected area designations (Priority 2). The remaining 82 CWR are well represented in SSSIs (Priority 3). Recommended actions for enhancing the conservation of each priority group of CWR are detailed in S1 Table. A further six CWR are listed under section 41 of the NERC act of rare and threatened species. These are wild asparagus (*Asparagus prostratus* Dumort.), upright goosefoot (*C*. *urbicum*), stinking goosefoot (*Chenopodium vulvaria* L.), least lettuce (*Lactuca saligna* L.), plymouth pear (*Pyrus cordata* Desv.) and sea barley (*Hordeum marinum*). Actions required to conserve all six of these taxa are outlined under the NERC act, however all actions are listed as ‘yet to start’ except for three associated with *P*. *cordata* which are ‘in progress’.

### *Ex situ* gap analysis

Sixty-five priority CWR (44%) have no *ex situ* accessions stored within UK gene banks. These taxa were assigned the highest level of priority (Priority 1). Amongst the taxa with no accessions are upright goosefoot (*C*. *urbicum*) and alpine cat’s-tail (*P*. *alpinum*), both of which are listed as Critically Endangered in England. Of the 83 CWR with accessions, perennial rye-grass (*Lolium perenne* L.) had the most (202). A further 22 CWR also had more than the advised minimum of five accessions [63,64] with next highest number of accessions belonging to plymouth pear (*P*. *cordata*) with 49. A total of 687 accessions exist for English priority CWR, 50% of which are stored at the Millennium Seed Bank, Kew. A total of 279 accessions are stored at the Genetic Resources Unit, IBERS at Aberystwyth University and a further 57 are stored at Warwick Genetic Resources Unit. Seven accessions lack holding institution data. The majority of accessions (92%) have fully georeferenced passport data. See S2 Table for the number of accessions stored in *ex situ* collections per priority CWR.

**Fig 3:**
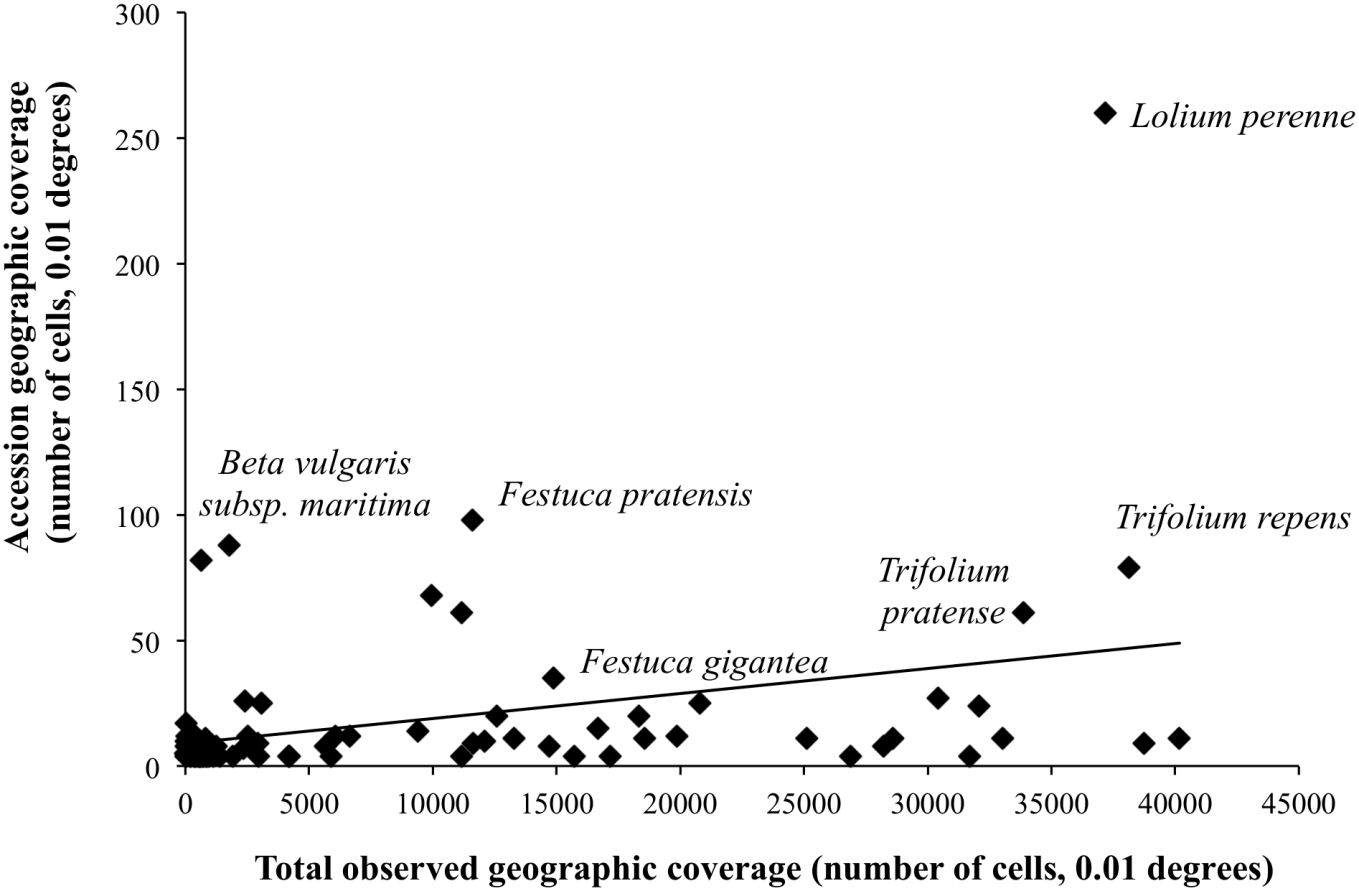
Geographic coverage of accession data. Taxa falling above the line show those with a GRS score higher than the mean GRS across all taxa, the accessions for these taxa cover a larger proportion of the taxon’s native range in England.

Geographical representativeness analysis revealed that only two taxa have a GRS score above the threshold of 30% [41]. These are two species with very restricted abundance and range in England, plymouth pear (*P*. *cordata*) and round-headed leek (*Allium sphaerocephalon* L.), suggesting that these are the only priority CWR for which *ex situ* collections are representative of their *in situ* range. The majority of taxa (69) had a GRS score below 5%. The relationship between the geographic coverage of *in situ* occurrence data and *ex situ* accession data is illustrated in Fig 3. It is clear that forage CWR tend to have higher numbers of accessions stored *ex situ* than food CWR, but also that for the majority of priority CWR taxa in England GRS percentages are extremely low. This indicates clear gaps in *ex situ* collections for priority CWR in England.

On combining the number of accessions and GRS results, it was found that only plymouth pear (*P*. *cordata*) is currently well represented in *ex situ* collections (i.e. assigned priority level 6). In addition, 22 CWR were listed in priority level 5 (having greater than five accessions but with a GRS lower than 30%). Over a third of CWR were listed as priority level 3 (36%) with between one and four accessions and a GRS below 30%. The majority of CWR were assigned to priority level 1 (44%), with no accessions. See S1 Table for a full list of assigned priority levels and Table 1 for recommended actions per priority level required to enhance *ex situ* collections.

## Discussion

### Prioritisation to create a CWR inventory

In England, conservation actions through Red Lists, site designations, species protection measures and other mechanisms have primarily been focussed upon nationally local and rare taxa. However this does not result in conservation of all CWR taxa and even less so the genetic diversity within them. For example, it has resulted in considerable efforts aimed at the conservation of wild asparagus (*A*. *prostratus*) [72,73] but little attention paid to conservation of potentially more important germplasm in taxa such as sea beet (*B*. *vulgaris* subsp. *maritima*) and cabbage (*Brassica oleracea* L.). Assessment and specific conservation planning for all CWR (food, forage, fodder, forestry, medicinal, industrial etc.) is therefore an important adjunct to more traditional conservation assessments and actions if CWR and their genetic diversity are to be adequately conserved. The English inventory of 148 priority CWR was developed based on criteria selected through consultation with Natural England. It identifies priorities for CWR conservation with a focus on the genetic resources most likely to be valuable to global food security. In addition to the current study, further work should be carried out to ensure comprehensive conservation of all other valuable plant-derived supplies and their wild relatives.

Identifying priority CWR based on meaningful criteria is the first step in planning for CWR conservation. Criteria selected to produce the English CWR inventory are similar to those selected in the development of CWR inventories in other countries. Three key criteria are most commonly cited: CWR native status, economic value of the related crop and degree of relatedness to an associated crop (Venezuela [30]; USA [32]; Finland [34] and Spain [35]), the latter two in particular showing the emphasis is frequently on criteria relevant to global food security. Other more distinct criteria have also been used. One such example can be seen in the CWR inventory for Cyprus where any CWR whose centre of diversity is within Cyprus, the Near East or the Middle East were prioritised, a decision taken through consultation with Cypriot stakeholders and focussing on conserving areas with the highest levels of CWR genetic diversity [37]. The selection of criteria can also be heavily influenced by available data. In England, the criterion ‘change in population range’ was included due the need to focus conservation efforts on CWR that are declining, and due to the availability of data looking at the change in distributions in plant species in Britain between two survey years (1987 and 2004) [57].

The selection and application of criteria for prioritisation of CWR is application specific and must be considered in terms of their degree of relevance to national and international conservation needs and priorities [74,75]. The process should involve key national stakeholders and conservation organisations to ensure their support for any conclusions drawn from such analysis but should equally consider whether the process addresses international policy targets e.g. Aichi target 13 [47]. Though the use of different criteria can change the pool of CWR listed as priority, if the criteria selected are appropriate to the project objectives and respond to policy then the various approaches should not compromise the achievement of effective conservation of CWR.

### *In situ* gap analysis

There are currently very few examples of active *in situ* conservation of CWR in the UK. Only six priority CWR in England (S1 Table) are recognised as threatened and are therefore listed on country or UK conservation priority lists [66,76] and just two of these six species have long-term and active conservation plans (wild asparagus–*A*. *prostratus* and plymouth pear–*P*. *cordata*) [72,73,77]. This shows there is a need for a concerted effort to enhance *in situ* conservation in England for CWR.

The results of the complementarity analysis revealed 15 grid squares across England that together are sufficient to conserve all priority English CWR, though for this to be possible active conservation of CWR populations and genetic diversity would need to be established within each of these sites. Each complementarity square overlaps with at least one protected area and over half of English priority CWR are well represented in SSSI protected areas. A further 22% are well represented in other protected area designations. Though presence within protected areas may offer a level of passive protection [78,79] it is important that there is specific monitoring and management for CWR to ensure their long-term survival. It may also be the case that the existing management of a protected area may conflict with the needs of the CWR, for example, on The Lizard Peninsula in Cornwall where there is concern over the impact of the level of cattle grazing in coastal sites on already threatened populations of wild asparagus (*A*. *prostratus*, Steve Townsend pers. comm.). This emphasises the need for incorporating active CWR conservation, ideally in the form of genetic reserves where conservation of genetic diversity is a priority, into existing protected area planning to enable CWR monitoring and appropriate management. Further analysis revealing the number of hectares of each protected area designation overlapping the 15 grid squares identified here has been carried out in 2015 by Natural England (unpublished data) with a view to justifying the inclusion of CWR in the management plans of existing protected area designations. This can be a highly effective method for achieving active CWR conservation as it avoids the high start-up costs of acquiring land for a new reserve and it may only require minimal adjustments to existing management plans [10]. Standards for the establishment of CWR genetic reserves have been outlined by Iriondo *et al*. [80].

The complementarity analysis method used in the current study [62] is a valuable tool that gives a broad picture of which sites in a country or region are suitable for *in situ* CWR conservation. It is important to note that the priority grid square boundaries do not denote the recommended outline of CWR genetic reserves, rather they indicate broad-scale areas which will require more detailed investigation and ground-truthing in order to identify more specific, fine-scale locations appropriate for *in situ* conservation of CWR both inside and outside of protected areas [34]. Part of this fine-scale selection of priority CWR populations and sites should consider the individual priority levels assigned to each CWR (S1 Table) but should also involve an assessment of the genetic diversity within target CWR [81,82]. With this data available, it ensures that conserved populations are representative of the range of genetic diversity that exists across a delineated area and that the selected populations are also complementary to one another. This is an ideal that has been achieved on the Lizard Peninsula in Cornwall where the range of genetic diversity in eight CWR was assessed and populations were identified as priority based on this data (Fielder *et al*., submitted).

Traditional conservation of rare and threatened plants generally takes place within designated protected areas. CWR however could also benefit from an approach that targets their conservation outside of protected areas. It is widely accepted that CWR tend to be associated with pre-climax communities and areas experiencing anthropomorphic change [10] and though such habitats are present within protected areas, habitats outside protected areas are likely to experience more stochastic change. This in turn could be exposing CWR to a range of different selection pressures, which could lead to adaptation of novel and potentially beneficial traits that could be exploited in crop development. A recent study by Jarvis *et al*. (submitted) has been able to provide strong evidence that UK CWR show preferences for linear landscape features such as field margins and road verges. Further, the current study revealed that 23% of priority English CWR are poorly represented within the existing protected area network. As such, it should be seen as a priority that alongside the establishment of genetic reserves for *in situ* conservation of CWR inside protected areas, there should be an additional focus on monitoring and management of CWR populations outside of protected areas. This approach would be most effective if undertaken with the approval and involvement of appropriate landowners and land managers.

### *Ex situ* gap analysis

There are clear gaps in *ex situ* CWR collections with just over half of the English priority CWR having any stored accessions at all. Of the taxa with accessions, very few have more than one. Ideally, it is recommended that at least five different populations are stored *ex situ* in gene banks to ensure that the collections represent the range of genetic diversity found within and among populations *in situ* [63]. From the results of the current gap analysis it is clear that this is a target of which English collections are currently falling short with only 16% of English priority CWR having greater than five stored accessions.

The GRS method for analysing the geographic coverage of *ex situ* accessions has been used successfully in previous studies [37,38,41], providing a broad picture of the representativeness of collections. In the case of England, it is apparent that most collecting effort has so far been focussed on forages. There has been a particular focus on the collection of perennial rye-grass (*L*. *perenne*) accessions at IBERS, Wales where extensive research has been carried out on this species due to its high importance as forage in temperate areas [83]. Ramírez-Villegas *et al*. [41] advise that a GRS result above a threshold of 30% suggests a CWR taxon is adequately conserved *ex situ* (though comparative genetic diversity studies are still required to validate this). Using GRS in combination with the total numbers of accessions per CWR provides a useful means of categorising CWR into priority levels to imply the urgency of further collecting.

In previous GRS studies herbarium specimens have been used to infer the full species distribution across a country or continent, providing a useful comparison of *in situ* distributions and gene bank representativeness [37,38]. In contrast, the use of herbarium specimens was not necessary in England due to the volume of field occurrence data available, directly showing species distributions across the country. However, the comparison of these occurrence records to the relatively few *ex situ* accessions suggests that extensive collecting would be necessary to achieve the 30% threshold for all CWR in England. In such cases where there is such a discrepancy between the volumes of available *in situ* and *ex situ* data, it may be more appropriate to consider each taxon on a case-by-case basis and to employ genetic or ecogeographic methodologies. In this way it would be possible to see more clearly whether further collecting is necessary.

*Ex situ* collections should be considered as a backup of material, which should be representative of populations conserved *in situ* [84]. In addition, it is necessary that CWR accessions are stored in gene banks to enable plant breeders and other users to access this material for use in crop improvement. Therefore, it is a prerequisite that *ex situ* collections represent the range of genetic diversity found within and among *in situ* CWR populations [85]. This can be achieved by proxy through collection of material from the full ecological and geographic range of each CWR, though direct analyses of genetic diversity using molecular markers will always be preferable and should be carried out where possible. The inclusion of genetic diversity and/or ecogeographic diversity data in gene bank databases would take large steps towards improving the completeness of *ex situ* CWR collections [35].

### Recommendations for enhancing CWR conservation in England

- The CWR inventory should be regularly reviewed (e.g. once every ten years) with the involvement of key stakeholders, providing the opportunity for: 1) national and international priorities to be reviewed according to policy, climate change and other factors such as pests and diseases, invasive species, pollution etc. 2) appropriate prioritisation criteria to be re-evaluated and 3) incorporation of more up-to-date data. Moreover, prioritisation of all other categories of CWR in England (not just those relating to human food or animal forage/fodder crops) should be undertaken.
- The *in situ* gap analysis results presented here should be used as a guide for enhancing CWR conservation within England. It is encouraged that existing protected areas (including, but not limited to SSSIs, SACs and NNRs) fully integrate CWR conservation into species and habitat management plans. Where possible these protected areas should strive to meet the agreed standards for CWR genetic reserves [80], particularly where they overlap with the 15 complementarity squares. Work is already underway on The Lizard Peninsula, Cornwall and in Purbeck, Dorset to achieve this.
- CWR conservation should also be encouraged outside of protected areas. Relevant landowners and land managers should be given the opportunity to agree to the quality standards for CWR genetic reserves, with the aim of establishing long-term CWR conservation. Governmental incentives (e.g. through the Rural Development Programme) could be provided to encourage this. Appropriate management of farmed semi-natural habitats such as hedgerows, grasslands and coastlands, as well as low intensity management of field margins and buffer strips would benefit CWR [9].
- To maximise the level of genetic diversity conserved *in situ* for priority CWR, at least five populations representing their full geographic range in England should be conserved per CWR. Where possible direct genetic analyses of populations of all priority CWR (in all categories) is encouraged. Initial focus should be on CWR assigned to *in situ* priority level 1 (S1 Table).
- Accurate records of the locations of all CWR throughout England are required, ideally along with assessments of population sizes and densities. All demographic data should be made available in an online database (e.g. NBN Gateway or BSBI distribution database). The data should be widely accessible and easy to update with new records.
- *Ex situ* collections need to be representative of *in situ* genetic diversity within and among populations of all CWR categories. To address this, there should be a renewed effort to improve the completeness of *ex situ* collections of English CWR by ensuring a minimum of five accessions representative of the geographic range of each CWR are stored in gene banks. Where possible genetic analyses should also be undertaken to achieve this goal. Initial focus should be on CWR in *ex situ* priority level 1 (S1 Table). This threshold is not intended to be prescriptive but should be a seen as a minimum, above which the specific life histories of taxa are also considered to ensure the maximum range of diversity is conserved within the accessions maintained *ex situ*.
- Existing accessions should also be assessed for regeneration ability and to ensure they are being maintained according to gene bank ‘best practices’ to enable long-term viability and use.
- Accessions held in long-term storage should be regularly updated with new material to capture the genotypic evolution of *in situ* populations over time.
- Accessions should have at least one duplicate stored in a geographically distant gene bank to reduce the likelihood of material being lost in any unforeseen circumstances.
- Accessions stored in gene banks should be made available to plant breeders for use in crop improvement, contributing to the development of new crop varieties.

## Conclusion

Conservation in England of the 148 CWR identified as priority in the context of food security is currently incomplete. This paper presents recommendations for enhancing their conservation based on both national and international priorities. The methodology described is applicable to all types of CWR (not only those with a role in improving food security) and can be used to achieve comprehensive coverage of all wild relatives. Through the integration of CWR into existing protected area management plans and establishment of genetic reserves in CWR hotspots, *in situ* CWR conservation can be simply but effectively improved. Together with a representative back up of material stored in gene banks this will provide effective, long-term monitoring and management of English CWR whilst facilitating their use in crop improvement. In this way, active and long-term conservation of English CWR can be established, contributing to European and global efforts to underpin future food security.

## Supporting Information

S1 Fig Economic value of English crops. Mean value of production at market prices (£ million) in England between 2007 and 2011 [55] for socio-economic crops with native or archaeophyte CWR occurring within England.
(TIF)

S2 Fig Complementarity analysis. The percentage of additional priority CWR contained within each of the 15 priority grid squares/candidate sites recommended for CWR genetic reserves.
(TIF)

S1 Table *In situ* and *ex situ* priority levels assigned to each priority English CWR. The criteria for each priority level and the recommended actions associated with each are also listed.
(XLSX)

S2 Table Number of accessions stored in gene banks for each of the 148 priority English CWR and their GRS values. (DOCX)
