# Supplementary figures and images for: Enhancing the Conservation of Crop Wild Relatives in England
Source: PLoS One. 2015 Jun 25;10(6):e0130804. doi: 10.1371/journal.pone.0130804 (PMC4481409; doi:10.1371/journal.pone.0130804)

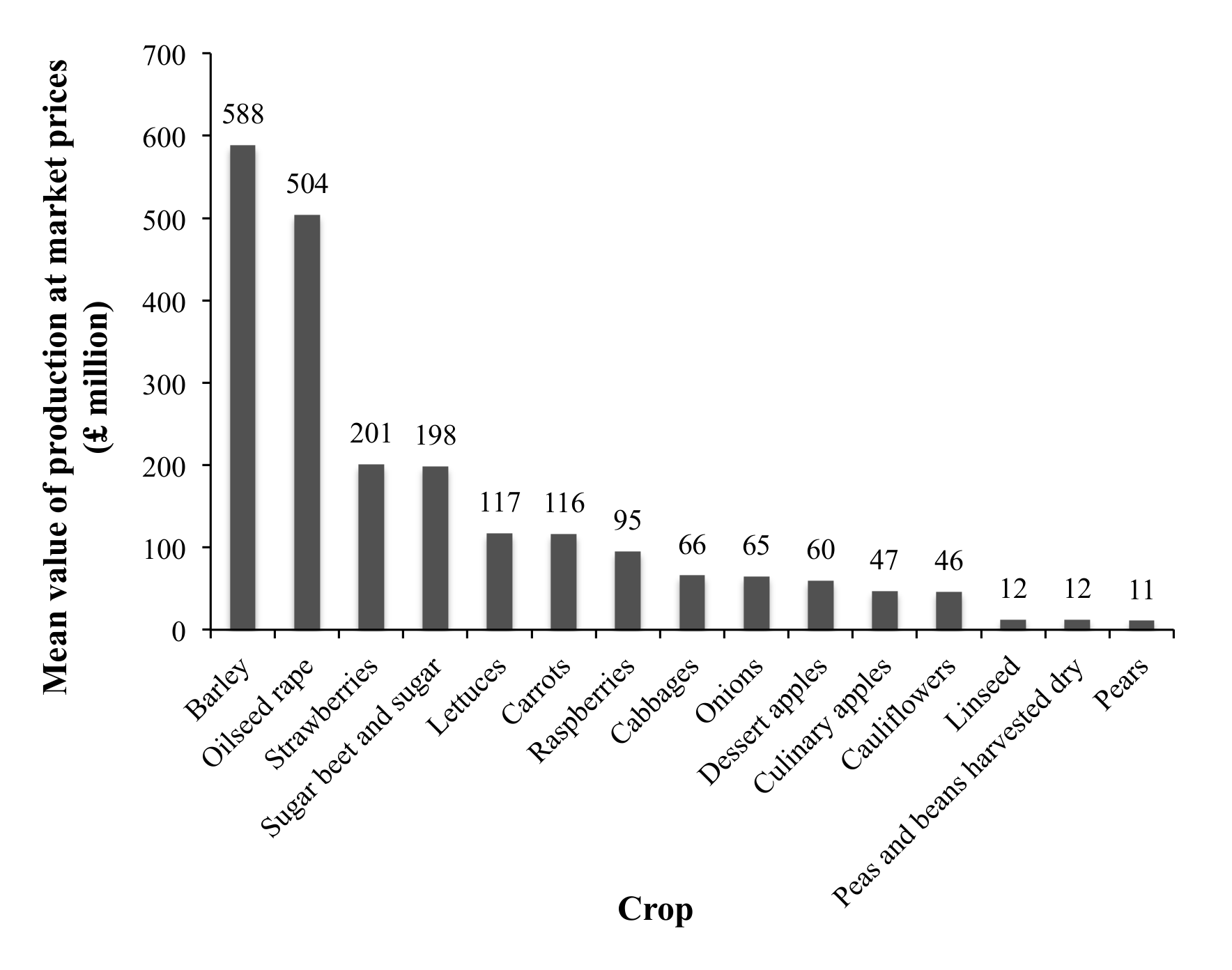

Supplement: S1 Fig — Mean value of production at market prices (£ million) in England between 2007 and 2011 [55] for socio-economic crops with native or archaeophyte CWR occurring within England. (TIF) [file pone.0130804.s001.tif]

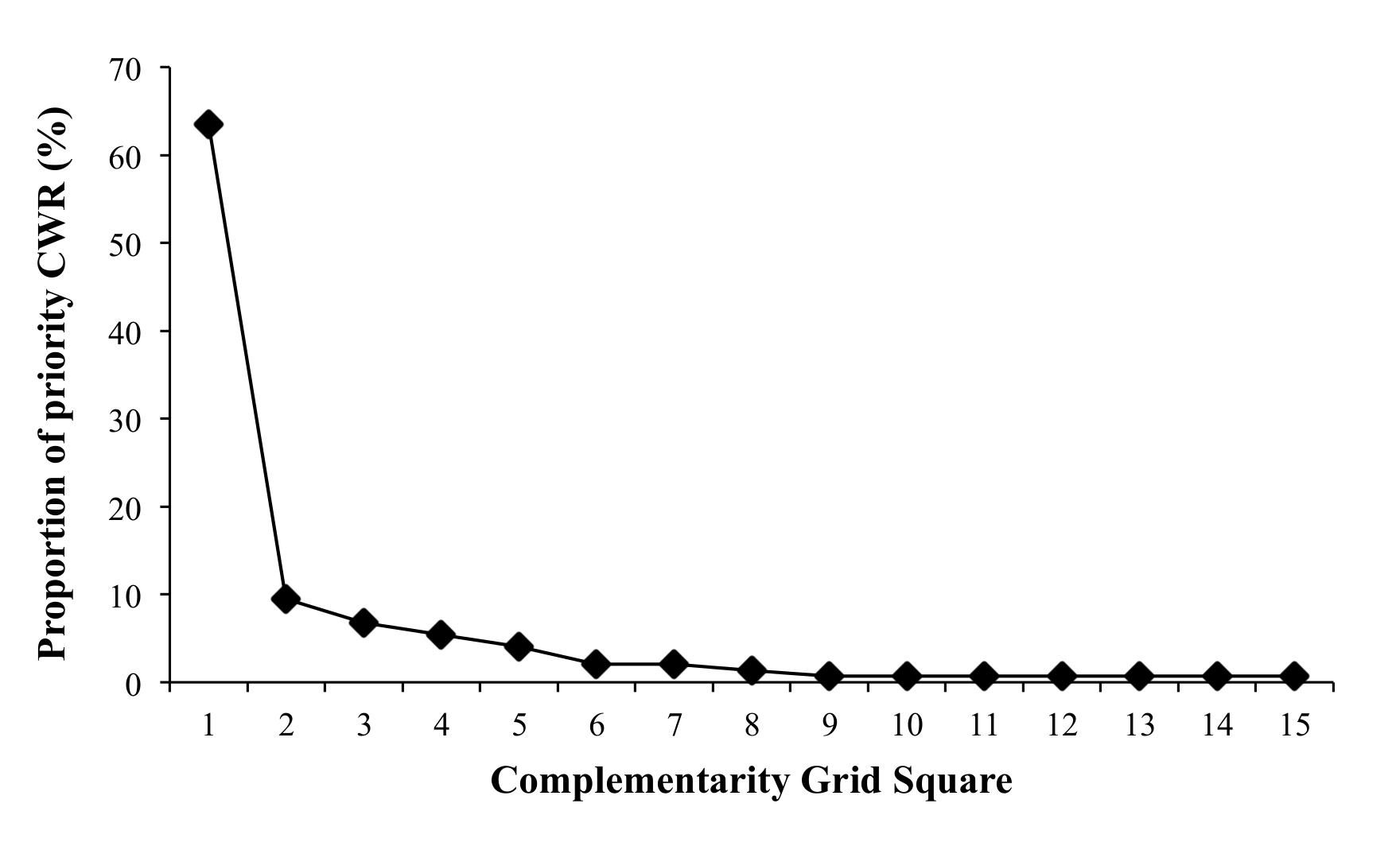

Supplement: S2 Fig — The percentage of additional priority CWR contained within each of the 15 priority grid squares/candidate sites recommended for CWR genetic reserves. (TIF) [file pone.0130804.s002.tif]
