# Supplementary material for: Enhancing the Conservation of Crop Wild Relatives in England
Source: PLoS One. 2015 Jun 25;10(6):e0130804. doi: 10.1371/journal.pone.0130804 (PMC4481409; doi:10.1371/journal.pone.0130804)
Supplement: S2 Table — (DOCX) [file pone.0130804.s004.docx]

| **Table S2** Number of accessions stored in gene banks for each of the 148 priority English CWR and their GRS values | | |
| --- | --- | --- |
| **CWR** | **Total Accessions** | **GRS (%)** |
| *Lolium perenne* | 202 | 0.70 |
| *Pyrus cordata* | 49 | 94.44 |
| *Trifolium repens* | 41 | 0.21 |
| *Brassica oleracea* | 40 | 12.71 |
| *Beta vulgaris* subsp. *maritima* | 30 | 4.97 |
| *Festuca pratensis* | 28 | 0.84 |
| *Daucus carota* subsp. *carota* | 21 | 0.55 |
| *Lactuca serriola* | 19 | 0.68 |
| *Trifolium pratense* | 18 | 0.18 |
| *Vicia sativa* subsp. *nigra* | 16 |  |
| *Lathyrus pratensis* | 13 | 0.09 |
| *Asparagus prostratus* | 11 |  |
| *Festuca gigantea* | 11 | 0.24 |
| *Lotus corniculatus* | 11 | 0.07 |
| *Lactuca virosa* | 8 | 1.08 |
| *Trifolium arvense* | 8 | 0.81 |
| *Linum perenne* subsp. *anglicum* | 7 | 4.26 |
| *Medicago lupulina* | 7 | 0.12 |
| *Vicia hirsuta* | 7 | 0.08 |
| *Fragaria vesca* | 6 | 0.11 |
| *Festuca arundinacea* | 5 | 0.05 |
| *Lactuca saligna* | 5 | 24.39 |
| *Linum catharticum* | 5 | 0.16 |
| *Raphanus raphanistrum* subsp. *maritimus* | 5 | 0.20 |
| *Rubus caesius* | 5 | 0.15 |
| *Anthoxanthum odoratum* | 4 | 0.04 |
| *Chenopodium vulvaria* | 4 | 19.35 |
| *Dactylis glomerata* | 4 | 0.03 |
| *Erodium cicutarium* | 4 | 0.18 |
| *Prunus avium* | 4 | 0.09 |
| *Sinapis arvensis* | 4 | 0.06 |
| *Allium sphaerocephalon* | 3 | 55.56 |
| *Allium ursinum* | 3 | 0.08 |
| *Chenopodium album* | 3 | 0.04 |
| *Chenopodium bonus-henricus* | 3 | 0.31 |
| *Holcus lanatus* | 3 | 0.02 |
| *Lotus pedunculatus* | 3 | 0.06 |
| *Malus sylvestris* | 3 | 0.08 |
| *Medicago polymorpha* | 3 | 1.34 |
| *Prunus spinosa* | 3 | 0.03 |
| *Trifolium fragiferum* | 3 | 0.48 |
| *Trifolium suffocatum* | 3 | 2.99 |
| *Allium ampeloprasum* | 2 | 21.05 |
| *Apium graveolens* | 2 | 0.64 |
| *Chenopodium glaucum* | 2 | 5.16 |
| *Daucus carota* subsp. *gummifer* | 2 | 4.90 |
| *Phalaris arundinacea* | 2 | 0.03 |
| *Phleum pratense* | 2 | 0.01 |
| *Prunus padus* | 2 | 0.14 |
| *Rubus chamaemorus* | 2 | 1.12 |
| *Vaccinium oxycoccos* | 2 | 0.30 |
| *Vicia lutea* | 2 | 2.93 |
| *Vicia parviflora* | 2 | 0.93 |
| *Allium oleraceum* | 1 | 0.46 |
| *Allium schoenoprasum* | 1 | 1.92 |
| *Allium vineale* | 1 | 0.07 |
| *Brassica nigra* | 1 | 0.10 |
| *Capsella bursa-pastoris* | 1 | 0.01 |
| *Chenopodium chenopodioides* | 1 |  |
| *Chenopodium hybridum* | 1 | 0.78 |
| *Chenopodium murale* | 1 | 1.07 |
| *Cichorium intybus* | 1 | 0.21 |
| *Hordeum marinum* | 1 | 0.47 |
| *Lathyrus sylvestris* | 1 | 0.55 |
| *Medicago minima* | 1 | 0.69 |
| *Medicago sativa* subsp. *falcata* | 1 | 0.66 |
| *Ornithopus perpusillus* | 1 | 0.13 |
| *Prunus domestica* subsp. *insititia* | 1 | 0.35 |
| *Ribes alpinum* | 1 | 1.14 |
| *Ribes rubrum* | 1 | 0.04 |
| *Ribes spicatum* | 1 | 1.93 |
| *Rubus idaeus* | 1 | 0.03 |
| *Trifolium bocconei* | 1 | 18.18 |
| *Trifolium glomeratum* | 1 | 0.61 |
| *Trifolium ochroleucon* | 1 | 0.29 |
| *Trifolium squamosum* | 1 | 0.71 |
| *Trifolium striatum* | 1 |  |
| *Trifolium strictum* | 1 | 11.43 |
| *Trifolium subterraneum* | 1 | 0.41 |
| *Trisetum flavescens* | 1 | 0.02 |
| *Vaccinium myrtillus* | 1 | 0.09 |
| *Vaccinium vitis-idaea* | 1 |  |
| *Vicia lathyroides* | 1 | 0.56 |
| *Agrostis canina* | 0 |  |
| *Agrostis capillaris* | 0 |  |
| *Agrostis curtisii* | 0 |  |
| *Agrostis gigantea* | 0 |  |
| *Allium scorodoprasum* | 0 |  |
| *Alopecurus geniculatus* | 0 |  |
| *Alopecurus pratensis* | 0 |  |
| *Apium inundatum* | 0 |  |
| *Arrhenatherum elatius* | 0 |  |
| *Asparagus officinalis* | 0 |  |
| *Atriplex glabriuscula* | 0 |  |
| *Atriplex portulacoides* | 0 |  |
| *Brassica rapa* subsp. *campestris* | 0 |  |
| *Calamagrostis epigejos* | 0 |  |
| *Chenopodium ficifolium* | 0 |  |
| *Chenopodium polyspermum* | 0 |  |
| *Chenopodium rubrum* | 0 |  |
| *Chenopodium urbicum* | 0 |  |
| *Corylus avellana* | 0 |  |
| *Cynosurus cristatus* | 0 |  |
| *Diplotaxis tenuifolia* | 0 |  |
| *Festuca ovina* subsp. *hirtula* | 0 |  |
| *Festuca ovina* subsp. *ophioliticola* | 0 |  |
| *Festuca ovina* subsp. *ovina* | 0 |  |
| *Hordeum murinum* subsp. *murinum* | 0 |  |
| *Hordeum secalinum* | 0 |  |
| *Koeleria macrantha* | 0 |  |
| *Lathyrus linifolius* | 0 |  |
| *Lepidium heterophyllum* | 0 |  |
| *Linum bienne* | 0 |  |
| *Medicago arabica* | 0 |  |
| *Medicago sativa* subsp. *varia* | 0 |  |
| *Melilotus altissimus* | 0 |  |
| *Pastinaca sativa* subsp. *sylvestris* | 0 |  |
| *Pastinaca sativa* subsp. *urens* | 0 |  |
| *Phleum alpinum* | 0 |  |
| *Phleum bertolonii* | 0 |  |
| *Poa annua* | 0 |  |
| *Poa nemoralis* | 0 |  |
| *Poa pratensis* | 0 |  |
| *Poa trivialis* | 0 |  |
| *Potentilla palustris* | 0 |  |
| *Prunus domestica* subsp. *domestica* | 0 |  |
| *Prunus domestica* subsp. *italica* | 0 |  |
| *Raphanus raphanistrum* subsp. *raphanistrum* | 0 |  |
| *Rorippa amphibia* | 0 |  |
| *Rorippa nasturtium-aquaticum* | 0 |  |
| *Rorippa sylvestris* | 0 |  |
| *Rubus saxatilis* | 0 |  |
| *Sinapis alba* subsp. *alba* | 0 |  |
| *Trifolium campestre* | 0 |  |
| *Trifolium incarnatum* subsp. *molinerii* | 0 |  |
| *Trifolium medium* | 0 |  |
| *Trifolium occidentale* | 0 |  |
| *Trifolium ornithopodioides* | 0 |  |
| *Trifolium scabrum* | 0 |  |
| *Vaccinium microcarpum* | 0 |  |
| *Vaccinium uliginosum* | 0 |  |
| *Vicia bithynica* | 0 |  |
| *Vicia cracca* | 0 |  |
| *Vicia orobus* | 0 |  |
| *Vicia sativa* subsp. *segetalis* | 0 |  |
| *Vicia sepium* | 0 |  |
| *Vicia sylvatica* | 0 |  |
| *Vicia tetrasperma* | 0 |  |
